# Action *relevance* in linguistic context drives word-induced motor activity

**DOI:** 10.3389/fnhum.2014.00163

**Published:** 2014-04-01

**Authors:** Pia Aravena, Mélody Courson, Victor Frak, Anne Cheylus, Yves Paulignan, Viviane Deprez, Tatjana A. Nazir

**Affiliations:** ^1^L2C2 Institut des Sciences Cognitives - Marc Jeannerod, CNRS/UCBL, Université Claude Bernard Lyon1Bron, France; ^2^Département de Kinanthropologie, Faculté des Sciences, Université du Québec à MontréalMontréal, Canada

**Keywords:** embodied language, context-dependency, lexical semantics, conceptual flexibility, situation models

## Abstract

Many neurocognitive studies on the role of motor structures in action-language processing have implicitly adopted a “dictionary-like” framework within which lexical meaning is constructed on the basis of an invariant set of semantic features. The debate has thus been centered on the question of whether motor activation is an integral part of the lexical semantics (embodied theories) or the result of a post-lexical construction of a situation model (disembodied theories). However, research in psycholinguistics show that lexical semantic processing and context-dependent meaning construction are narrowly integrated. An understanding of the role of motor structures in action-language processing might thus be better achieved by focusing on the linguistic contexts under which such structures are recruited. Here, we therefore analyzed online modulations of grip force while subjects listened to target words embedded in different linguistic contexts. When the target word was a hand action verb and when the sentence focused on that action (John **signs** the contract) an early increase of grip force was observed. No comparable increase was detected when the same word occurred in a context that shifted the focus toward the agent's mental state (John **wants** to *sign* the contract). There mere presence of an action word is thus *not sufficient* to trigger motor activation. Moreover, when the linguistic context set up a strong expectation for a hand action, a grip force increase was observed even when the tested word was a pseudo-verb. The presence of a known action word is thus not *required* to trigger motor activation. Importantly, however, the same linguistic contexts that sufficed to trigger motor activation with pseudo-verbs failed to trigger motor activation when the target words were verbs with no motor action reference. Context is thus not by itself sufficient to supersede an “incompatible” word meaning. We argue that motor structure activation is part of a dynamic process that integrates the lexical meaning potential of a term and the context in the online construction of a situation model, which is a crucial process for fluent and efficient online language comprehension.

## Introduction

A growing number of evidence supports the idea that the brain's motor structures are implicated in the processing of language referring to motor actions (for a review see Hauk and Tschentscher, 2013). However, the crosstalk that the neural networks underlying motor actions entertain with language processes is not well understood. Currently, the theoretical approaches that aim at accounting for the role of motor activation during action-language processing mainly focus on the question of whether language-induced motor activity should be considered as an integral part of lexical semantics or, rather, as resulting from ensuing “higher-level” processes involved in the construction of mental representations of the described state of affairs (Hauk et al., 2008a,b; Van Elk et al., 2010; Bedny and Caramazza, 2011). Answering this question is believed to solve the issue of whether motor activation is relevant for action-language processing or merely an epiphenomenon (for reviews on the theoretical accounts in this debate, see Meteyard et al., 2012; Pulvermüller, 2013). However, determining whether language-induced motor activation is part of one of these two processes implies considering lexical meaning access and the representation of the situation described by the context as separated processes. Such a dichotomic view, however, is grounded in models of lexical meaning representation currently regarded as no longer tenable (Hoenig et al., 2008; Raposo et al., 2009; see also Egorova et al., 2013). A better understanding of language-induced motor activity may thus require a shift in theoretical perspective.

Research on the role of language induced sensorimotor activation has generated a large body of sometimes conflicting experimental results (see e.g., Hauk et al., 2004 vs. Postle, McMahon, Ashton et al., 2008; Buccino et al., 2005 vs. Pulvermuller et al., 2005; for a review see Willems and Francken, 2012). While these inconsistencies could be seen as an obstacle for the understanding of the crosstalk between language and motor structures, they could alternatively be regarded as providing important insights into the nature of this phenomenon: the heterogeneity in the findings could well indicate that the recruitment of sensorimotor structures crucially depends on the linguistic and extra-linguistic context (see Hoenig et al., 2008; Sato et al., 2008; Papeo et al., 2009, 2012; Rueschemeyer et al., 2010; Mirabella et al., 2012; Tomasino and Rumiati, 2013; for a recent review, see Yang, 2013; see also van Dam et al., 2011; Willems and Casasanto, 2011). That the context a word is uttered in partially determines its meaning is well established among linguists and psycholinguists (e.g., Allwood, 2003; Elman, 2011). According to Allwood (2003) for instance, lexical meaning representations emerge from multiple interactions within a broad knowledge structure. This word knowledge, that Allwood refers to as the “meaning potential” of a word, comprises the set of all the information that the word has been used to convey either by an individual or by a language community. Within the bounds of this meaning potential, the kind of event, property, or entity a given word is taken to denote shift according to the context the word occurs in.

In line with the above view, a vast number of psycholinguistic studies have demonstrated early effects of context on lexical semantics processing (for a review, see Spivey and Huette, 2013). For example, Federmeier et al. (2007) recorded ERPs as participants read target words in weakly constraining (e.g., “Mary went into her room to look at her gift”) or strongly constraining (e.g., “The child was born with a rare gift”) sentence contexts. The authors analyzed the N400 ERP-component, whose magnitude is positively correlated to interpretative problems, and found a smaller N400 for the same target words in the strongly compared to the weakly constraining contexts. The brain thus seems to use context information to generate likely upcoming stimuli and to prepare ahead of time for their processing (see also Kako and Trueswell, 2000; Kamide et al., 2003; Chambers and Juan, 2008; Bicknell et al., 2010). Note that this “lexical anticipation” phenomenon involves evaluating the contextual properties of a word and not merely its characteristics as an entity of the mental lexicon. The whole event evoked when processing a sentence within a given context restricts the set of potential word referents (Kako and Trueswell, 2000; Kamide et al., 2003; Chambers and Juan, 2008; Bicknell et al., 2010; Kukona et al., 2011). In other terms, lexical meaning access profits from a representational state of the situation described by the context (e.g., Nieuwland and Van Berkum, 2006; Hagoort and van Berkum, 2007; Metusalem et al., 2012). This representational state, which can assimilate information about time, social relations, mental acts, space, objects, and events (MacWhinney, 2005; Frank and Vigliocco, 2011), has been termed by linguists and philosophers as “mental models” or “situation model” (Johnson-Laird, 1983; Van Dijk and Kintsch, 1983; Zwaan and Radvansky, 1998; Zwaan and Madden, 2004). As demonstrated by Nieuwland and Van Berkum (2006), situation models can even overrule constraints provided by core lexical-semantic features such as animacy, which, in classic linguistic semantics, is encoded in the mental lexicon. Hence, when participants listened to a story about a dancing peanut that had a big smile, the canonical inanimate predicate “salted” for the inanimate object “peanut” elicited a larger N400 component than the animate predicate “in love.” Situation models can thus neutralize processing difficulties due to animacy violations, confirming that lexical meaning does not necessarily involve an initial context-independent semantic computation.

Despite the remarkable body of evidence regarding the context dependency of lexical meaning, these results have rarely been taken into account in the cognitive neuroscience literature that discusses the role of motor structures in action-language processing. In fact, many researchers in this domain seem to have implicitly relied on theoretical views that apprehend word recognition and semantic processing in a form-driven, exhaustive, bottom-up fashion (Swinney and Love, 2002; MacDonald and Seidenberg, 2006). In this manner, semantic and pragmatic context exerts its effects only after word meaning has been elaborated. What is more, it seems as if it is tacitly assumed that words have fixed meanings that are accessed like entries in a dictionary (c.f. “conceptual stability”; Hoenig et al., 2008. See also Elman, 2011). However, within a theoretical frame that considers lexical meaning access as an interactive process, integrating information from many different sources, the question of whether language-induced motor activation is an integral part of lexical meaning or a mere effect of the ensuing construction of a situation model (Hauk et al., 2008a,b; Chatterjee, 2010; Bedny and Caramazza, 2011) does not make sense. Therefore, this issue will not satisfactorily inform the main interrogation regarding the function of motor activation in action-language processing. We believe that an understanding of the role of motor structures in the construction of linguistic meaning requires a detailed exploration of the context under which motor structures are recruited during action-language processing.

Critical results along this line were provided by Taylor and Zwaan (2008). These authors demonstrated that in a sentence describing a manual rotation (e.g., “He placed his hand on the gas cap, which he opened slowly”), compatible motor responses (i.e., manual rotation of a knob in a congruent direction with the linguistically described activity) are facilitated during reading the verb “opened.” Motor responses are also facilitated while reading of the adverb that modifies the action verb (i.e., “slowly”), but not while reading of the adverbs that modify the agent (e.g., “He placed his hand on the gas cap, which he opened *happily*”). According to Taylor and Zwaan (2008), the difference between the two conditions is explained by the fact that the adverbs that modify the action maintain the linguistic semantic focus on the action described in the sentence. Note that these results suggest that motor structure activation is sustained beyond the lexical-entity of the action term, extending to the broader linguistic event in which the word is embedded. Results from our laboratory further support this view. By analyzing online grip force variations that index cerebral motor activity in response to target words (c.f. Frak et al., 2010), our study revealed an increase of grip force starting around 200 ms after the onset of a manual action word when the word occurred in an affirmative sentence (e.g., “Fiona lifts the luggage”), but not when it occurred in a negative sentential context (“Fiona does *not* lift the luggage”) (Aravena et al., 2012). Our interpretation of these data is that in affirmative context, motor features of the target word are activated because of the *relevance* of the action within the situation model. In negative contexts the motor features remain irrelevant in spite of the actual presence of the action word in the sentence, because the sentence-induced situation model does not focus on the action.

In the present study, we present two experiments that further investigate how the sentential context modulates word-induced motor activation. As in our previous studies (Frak et al., 2010; Aravena et al., 2012), we measured grip force variations while subjects listen to words that describe manual motor actions. Note that an increase of word-induced grip force can be interpreted as an incomplete inhibition of the output of primary motor cortex activity (Jeannerod, 1994; Frak et al., 2010). No motor task associated to the linguistic process was required, as participants were asked to count how many sentences contain a name of a country. This ensured the ecology of the experimental environment as it simulates a quite natural linguistic situation.

In Experiment 1 we set out to investigate the effect of linguistic focus on action-verb induced motor activity by making use of the *volition modality* (“want to do,” see Morante and Sporleder, 2012). Volition is a grammatical modality that pertains to the intentions of an agent with respect to an action. It sets an action in an *irrealis mood* indicating that the relevant situation or action has not yet happened. Indeed, wanting to do X presupposes that X is not currently being done or taking place. Hence, the situation model evoked by the volition modality does not focus a motor action. In Experiment 2 we assessed the degree of context-dependency of language-induced motor activation by measuring motor activity at the point where the target word is expected. For example, for an utterance beginning with “With his black pen, James… ” the word “writes” is a continuation that is far more likely than the word “walk,” as the former evokes a more plausible action for the use of the “black pen” (see Bicknell et al., 2010; Matsuki et al., 2011). To investigate the anticipatory effects of an action context on the subsequent word processing, we used either a pseudo-verb with no associated reference or a verb whose associated reference was incompatible with the action meaning anticipated by the context. In keeping with the findings of our experiment with negative contexts, we predicted that the processing of an action word should neither be sufficient nor even necessary to activate motor structures. Hence:

- An action word (e.g., to soap) embedded in a volitional sentence whose focus is on the mental state of the agent (i.e., “Jamal **wants** to *soap* his dirty shirt”) should not trigger an increased grip force.
- In a context that primes properties of a hand-action verb, a pseudo-verb (e.g., “With his black pen, Paul **griles** the contract”) should suffice to trigger an increase in grip force. However, given that contextual parameters are actualized rapidly by incoming words, contextual cues that could otherwise trigger motor activity should fail to do so if the ensuing verb is not compatible with the anticipated action meaning (e.g., “With his black pen, Paul **plans** to sign the contract”).

## Materials and methods

### Experiment 1: volition

#### Ethics statement

All of the participants in this study gave an informed written consent. The study was approved by the Ethical Committee CPP (Comité de Protection des Personnes) Sud-Est II in Lyon, France.

#### Participants

All of the participants were French undergraduate students (18–35 years old; mean age = 21.7, *SD* = 1.5) and right-handed Edinburgh handedness inventory (Oldfield, 1971), with normal hearing and no reported history of psychiatric or neurological disorders. Twenty-five participants (including 13 females) participated in this study. Eight participants were eliminated from the analysis due to an extremely weak signal throughout the experiment, thus preventing the capture of grip-force. We used a grip-force mean below 0.13 V in combination with the absence of signal changes throughout the experiment as criteria for discarding participants from the analyses.

#### Stimuli

A total of 115 French sentences served as stimuli (see Supplementary Material). Ten were distractor-sentences containing a country name. The data from the trials using the distractor-sentences were not included in the analysis. Thirty-five target-action words were embedded into action-in-focus and volition-in-focus sentences resulting in 70 total sentences corresponding to the two conditions of the experiment: the action-in-focus and the volition-in-focus condition. All of the target action words were verbs denoting actions performed with the hand or arm (e.g., scratch or throw). Thirty-five sentences containing common nouns denoting concrete entities with no motor associations were used for comparison with earlier studies (e.g., Frak et al., 2010; Aravena et al., 2012). The target nouns and verbs were controlled for frequency, number of letters, number of syllables and bi- and trigram frequency (New et al., 2001, see Supplementary Material). Three examples of experimental stimuli are provided in Table 1.

**Table 1:** **Example of stimuli used in the Experiment 1 and their approximate English translation**.

**Condition**	**Sentence**	**English approximate translation**
Action-in-focus	Dans la salle de sport, Fiona **soulève** des haltères.	*At the gym, Fiona lifts the dumbbells*.
Volition-in-focus	A l'intérieur de l'avion, Laure **veut** soulever son bagage.	*In the plane, Laure **wants** to lift her luggage*.
Nouns	Au printemps, Edmonde aime le bosquet de fleur de son jardin.	*In the spring, Edmonde loves the flower-bush in her garden*

Underlined words represent the target words. Words in bold type represent the linguistic focus of the sentence.

All critical verbs were in the present tense and in neutral 3rd person. Verbs always occurred in the same position of the sentence. The sentences were spoken by a French male adult. His voice was recorded using Adobe Soundbooth and the recordings were adjusted to generate similar trial lengths using the Audacity 1.2.6 software. Two pseudo-randomized sentences lists were generated from trials; these lists contained uniform distributions of the different sentence types. The two lists were alternated between participants. The mean word duration was 459 ms (*SD* = 97 ms) for the nouns and 415 ms (*SD* = 78 ms) for the verbs. There was an interval of 2000 ms between the sentence presentations.

#### Equipment and data acquisition

**Figure 1:**
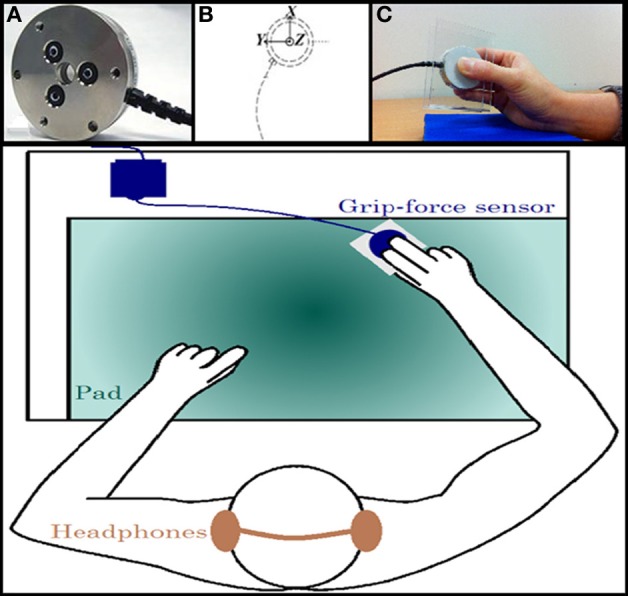
**Experimental material and setting. (A)** A standalone 6-axis load cell of 68 g was used (ATI Industrial Automation, USA). **(B)** The three main forces were recorded: Fx, Fy, and Fz as the longitudinal, radial and compression forces, respectively. **(C)** Participants hold the grip-force sensor in a precision grip with their right hand. Bottom panel: participants wore headphones and were comfortably seated behind a desk on which a pad was placed. They were asked to rest their arms on the pad, holding the sensor.

Two distinct computers were used for data recording and stimulus presentation to ensure synchronization between audio files and grip-force measurements (estimated error <5 ms). The first computer read the play-list of the pseudo-randomized stimuli. The second computer received two triggers from the first computer, which indicated the beginning and the end of the play-list. This second computer also recorded the incoming force signals from the load cell at a high sampling rate of 1 KHz. To measure the activity of the hand muscles, a standalone 6-axis load cell of 68 g was used (ATI Industrial Automation, USA, see Figure 1). In the present study, force torques were negligible due to the absence of voluntary movement; thus, only the three main forces were recorded: Fx, Fy, and Fz as the longitudinal, radial and compression forces, respectively (Figure 1B).

#### Procedure

Participants wore headphones and were comfortably seated behind a desk on which a pad was placed. They were asked to rest their arms on the pad, holding the grip-force sensor in a precision grip with their right hand (see Figure 1). The thumb, index, and middle fingers remained on the load cell throughout the experiment. Holding the sensor with the index, thumb, and middle finger implies more stability of the object (i.e., less grip force variations due to finger adjustments) than holding it with the index and thumb only.

The Experimenter demonstrated how to hold the grip sensor and participants were requested to hold the cell without applying voluntary forces.

The cell was suspended and not in contact with the table. The participants kept their eyes closed for the duration of the experiment. They were verbally instructed to listen to the spoken sentences. Their task was to silently count how many sentences contained the name of a country. To avoid muscular fatigue, a break of 10 s was given every 3 min. The total length of the experiment was 12 min.

#### Data analysis

Prior to the data analysis, each signal component was pretreated with the Brain Vision Analyzer 2.0 software (Brain Vision Analyzer software, Brain Products GmbH, Munich, Germany). The data were filtered at 10 Hz with a fourth-order, zero-phase, low-pass Butterworth filter, and a notch filter (50 Hz) was applied in case that artifact caused by electrical power lines would have persisted. Finally, a baseline correction was performed on the mean amplitude of the interval from −400 to 0 ms prior to word onset. The baseline correction was implemented because of a possible global change in grip-force during the session (12 min), and because we are only interested in grip-force changes. Thus, we adjusted the post-stimulus values by the values present in the baseline period. A simple subtraction of the baseline values from all of the values in the epoch was performed. As the participants were asked to hold the grip-force sensor throughout the experiment, a “negative” grip-force refers to a lesser grip-force and not to the absence of grip-force, which is impossible in this context. Only Fz (compression force) was included in the analysis as this parameter was determined to be the most accurate indicator of prehensile grip-force. The Fz signals were segmented offline into 1200 ms epochs spanning from 400 ms pre-stimulus onset to 800 ms post-stimulus. The segments with visually detectable artifacts (e.g., gross hand movements) and the trials that showed oscillations exceeding the participant's mean force were isolated and discarded from the analysis. A mean of 6.04 segments (17.2%) were discarded per condition. The Fz signals for action words in action-in-focus, action words in volition-in-focus and nouns were averaged for each participant and the grand mean was computed for each condition.

We selected three time windows (i.e., 100–300, 300–500, and 500–800 ms after word onset) that were identified as critical phases during the processing of words in auditory sentences in Friederici's (2002) model and that were used previously in our work for language-induced grip-force analysis (Aravena et al., 2012). Given that the conduction time between the primary motor cortex (M1) and hand muscle is approximately 18–20 ms (estimations using TMS, Rossini et al., 1999), we added 20 ms to each of these windows, resulting in 120–320 ms for the first window, 320–520 ms for the second time window and 520–800 ms for the third.

For each condition, the averaged grip-force values in the three time windows were compared with their proper baseline (i.e., averaged grip-force values over the segment between −400 and 0 ms before target word onset) using a one-sample *t*-test against zero; for a window that presented significant grip-force modulations with respect to the baseline, a comparison between the conditions was performed using repeated measures of Analysis of Variance (ANOVA). *Post-hoc* two-by-two comparisons were performed using the Bonferroni test. Since statistical significance is heavily dependent upon sample size, and our study sample was smaller than 20, we also report “effect sizes” (Cohen's *d*; Cohen, 1988). An effect size is calculated by taking the difference of the mean between two conditions and dividing this difference by the pooled standard deviation of the two conditions. This allows estimating how many standard deviations difference there is between the conditions. According to Cohen (1988) and effect size of.20 (i.e., a difference of a fifth of the standard deviation) is a small effects size. A medium effect size is 0.50 and a large effect size is 0.80.

### Experiment 2: pseudo-verbs

#### Ethics statement

All participants in this study gave an informed written consent. The study was approved by the Ethical Committee CPP (Comité de Protection des Personnes) Sud-Est II in Lyon, France.

#### Participants

All of the participants were French undergraduate students (18–35 years old; mean age = 21.7, *SD* = 2.1) and right-handed [Edinburgh Inventory definition (Oldfield, 1971)], with normal hearing and no reported history of psychiatric or neurological disorders. Nineteen subjects (including 10 females) participated in this study and none had participated in Experiment 1.

#### Stimuli

A total of 158 French sentences served as stimuli (see Supplementary Material). Ten were distractor-sentences containing a country name. The data from the trials using the distractor-sentences were not included in the analysis.

For this experiment, 37 pseudo-verbs were created obeying French's phonotactic constraints using the «Lexique Toolbox» of the data base Lexique 3 (New et al., 2001). The soundness of the verb as a French verb was controlled (see Supplementary Material). Thirty-seven target non-action words were utilized. All non-action words were verbs denoting no action performed with the hand or arm (e.g., decide, think), as confirmed by the stimuli validation process (see Supplementary Material). Thirty-seven target action words were included. All action words were verbs denoting actions performed with the hand or arm (e.g., scratch or throw) as established by the stimuli validation process (see Supplementary Material).

All the target words were controlled for frequency, number of letters, number of syllables, and bi- and trigram frequency (New et al., 2001).

The 37 action verbs, the 37 pseudo-verbs, and the 37 non-action verbs were embedded into action contexts. The 37 target non-action verbs were also embedded into non-action contexts.

Action contexts were designed in such a way that the first adverbial phrase and the subject of the sentence coded a situation, which anticipated a hand action. The degree of effector specificity (i.e., hand action) of action contexts and the action verb cloze probability were controlled. The “degree of effector specificity” was defined as how representative of a hand action was the action encoded by the sentence. All actions encoded by sentences were highly prototypical as hand actions. Cloze probability was defined as how easy was to anticipate a hand action verb from the previous sentential context. Only the contexts that induce highly cloze probability of hand action verbs were considered as action contexts (see Supplementary Material).

In summary, the present study exploited four conditions:

- action _context_ action_verb_ condition (action verb in action context)
- action _context_ pseudo_verb_ condition (pseudo-verb in action context)
- action _context_ non-action_verb_ condition (non-action verb in action context)
- non-action _context_ non-action_verb_ condition (non-action in non-action context).

Four examples of experimental stimuli are provided in Table 2.

**Table 2:** **Example of stimuli used in the Experiment 2 and their approximate English translation**.

**Condition**	**Sentence**	**English approximate translation**
Action_context_	Avec son stylo noir, Paul signe le contrat	*With his black pen, Paul signs the contract*
Action_verb_		
Action_context_	Avec son stylo noir, Paul grile le Contrat	*With his black pen, Paul griles the contract*
Pseudo_verb_		
Action_context_	Avec son stylo noir, Paul projette de signer le contrat	*With his black pen, Paul plans to sign the contract*
Non-action_verb_		
Non-action_context_	Une fois de plus, Thomas songe à rassembler toute la famille	*One more time, Thomas dreams to assemble all the family*
Non-action_verb_		

Underlined words represent the target words.

All critical verbs were in the present tense and in neutral 3rd person. Verbs always occurred in the same sentential position (see Table 2). The sentences were spoken by a French female adult. Her voice was recorded using Adobe Soundbooth and the recordings were adjusted to generate similar trial lengths using the Audacity 1.2.6 software. Three lists of 37 action contexts (A, B, and C) were created to avoid context repetition between the three action context conditions. Action words were included in A, when pseudo-verbs were included in B and non-action words in C, and they were included in B when pseudo-verbs were in C and non-action in A, etc. Therefore, three pseudo-randomized sentences lists were generated from such balanced combination (ABC, BCA, CBA) in addition to the non-action C-non-action V list and the 10 country sentences. These lists contained uniform distributions of the different sentence types. The three lists were alternated between participants. The mean word duration was 459 ms (*SD* = 97 ms). There was an interval of 2000 ms between the sentence presentations.

#### Equipment and data acquisition

The equipment and data acquisition from Experiment 1 were used in Experiment 2 (see also Aravena et al., 2012).

#### Procedure

The procedure from Experiment 1 was repeated with the exception that in the current experiment prior to the beginning of test participants were verbally instructed to apply a specific minimal force on the cell (i.e., between 0.08 and 0.13 V; that was surveyed by the experimenter in the visual signal online registration software) and maintain it throughout all the experiment without applying other voluntary forces. This instruction served to assure the operative capture of the signal, insofar as an extremely weak signal prevents the detection of grip-force variations as shown in Experiment 1 (from which eight participants were eliminated due to frail signals). The total length of the experiment was 18 min.

#### Data analysis

The analysis used for Experiment 2 was the same used in Experiment 1.

## Results

### Results experiment 1: volition

**Figure 2:**
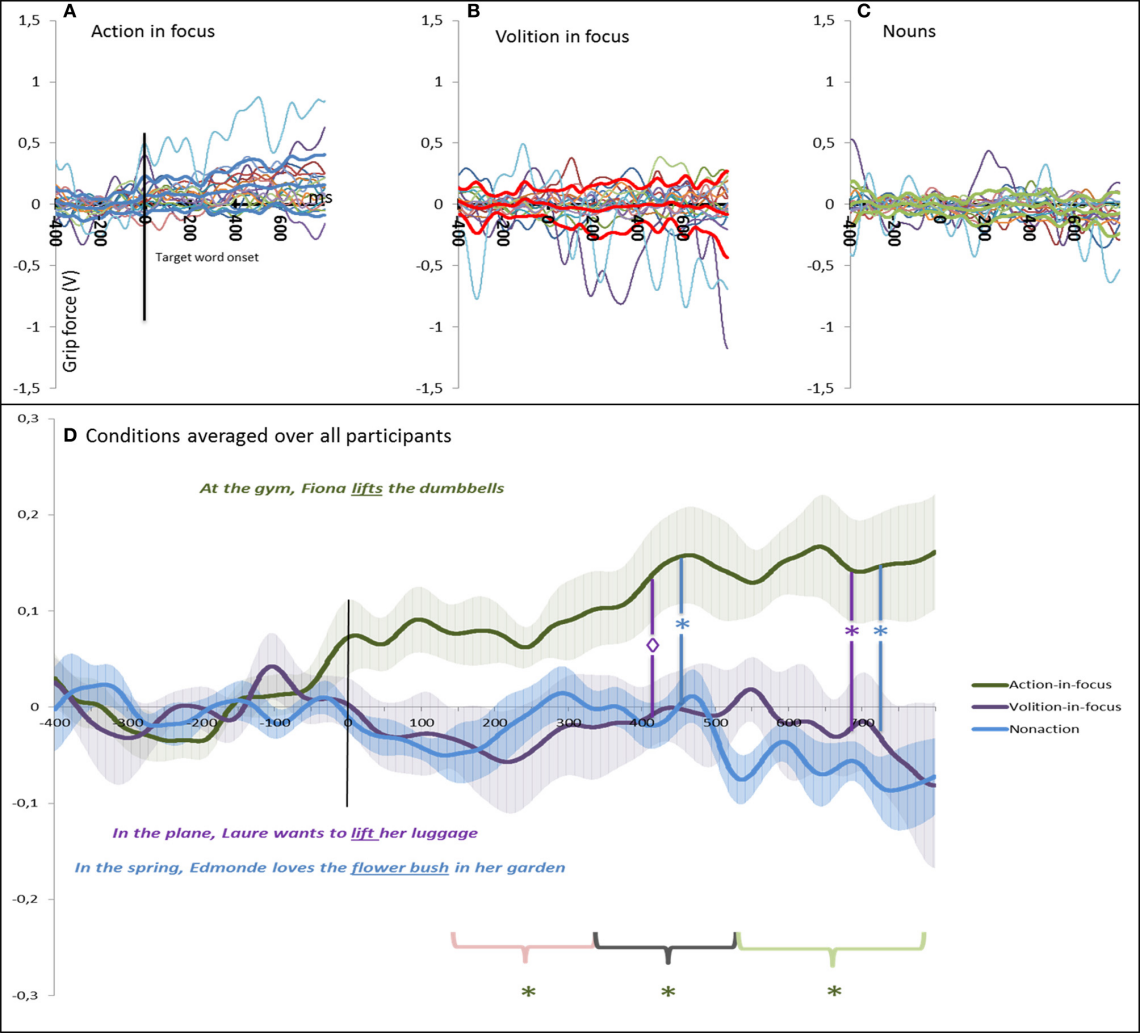
**Modulation of the grip-force amplitude as a function of time after target onset in Experiment 1 (Volition)**. **(A–C)** displays individual data for the three conditions (the bold lines represent the means and standard deviations) and **(D)** compares data of the three conditions averaged over all participants. In **(D)** we also show the standard error of the mean (s.e.m.) around the mean value across the subjects (shaded regions). For the action-in-focus condition a significant increase in the grip force was observed soon after target words presentations and it is maintained over the three intervals. This enhanced grip-force is significantly different from the volition condition in the two last windows and from the nouns conditions in the last window. The color of the asterisk refers to the color of the condition that is compared.

Figure 2 plots the variations in grip-force amplitude as a function of time after target word onset for the three experimental conditions (volition-in-focus condition, action-in-focus condition, and nouns condition). The top panel displays individual data for the three conditions and the bottom panel compares data of the three conditions averaged over all participants. As is obvious from the figure, for the action-in-focus condition a steady increase in the grip force [the compression force component of the load cell (Fz)] was observed soon after target words presentations and it is maintained until the last interval. By contrast, the volition and the nouns condition remained nearly constant at baseline.

For the action-in-focus condition the test against the baseline revealed a significant increase in the grip-force in the three time windows [*p* = 0.013, *p* = 0.009, *p* = 0.005 for 120–320, 320–520, 520–800 ms respectively]. No significant effects against baseline were observed for the volition-in-focus or for the nouns condition.

The ANOVA revealed significant effects of the conditions in the last two time windows [*F*_(2, 32)_ = 3.4505, *p* = 0.043 and *F*_(2, 32)_ = 5.6477, *p* = 0.007 respectively]. *Post-hoc* comparison (Bonferroni) for the second window showed that the Action condition (*M* = 0.08 V, *SD* = 0.1) differed significantly from the Volition condition (*M* = −0.01 V, *SD* = 0.1) [*p* = 0.05] and just failed to be significantly different from the Noun condition (*M* = −0.009 V, *SD* = 0.08) [*p* = 0.06 ns]. In the last window *post-hoc* comparison revealed that the Action condition (*M* = 0.14 V, *SD* = 0.19) different from the Volition condition (*M* = −0.02 V, *SD* = 0.18) [*p* = 0.02] as well as from the Noun condition (*M* = −0.03 V, *SD* = 0.8) [*p* = 0.007]. Table 3 summarizes the effect sizes (Cohen d) of the different comparisons. In all time windows large effect sizes were found for the difference between the Action vs. Nouns conditions as well as between the Action vs. Volition conditions.

**Table 3:** **Cohen's d for the differences between the various conditions in the three time windows**.

	**Nouns**	**Volition**
**TIME WINDOW 120–320 ms**		
Action	0.92	0.78
Volition	0.13	
**TIME WINDOW 320–520 ms**		
Action	0.99	0.76
Volition	0.08	
**TIME WINDOW 520–800 ms**		
Action	1.26	0.92
Volition	0.08	

All together these analyses confirm that the same action words embedded in sentences whose focus is on the mental state of the agent do not increase grip force in the same way as when they are embedded within sentences that focus the action.

### Results experiment 2: pseudo-verbs

**Figure 3:**
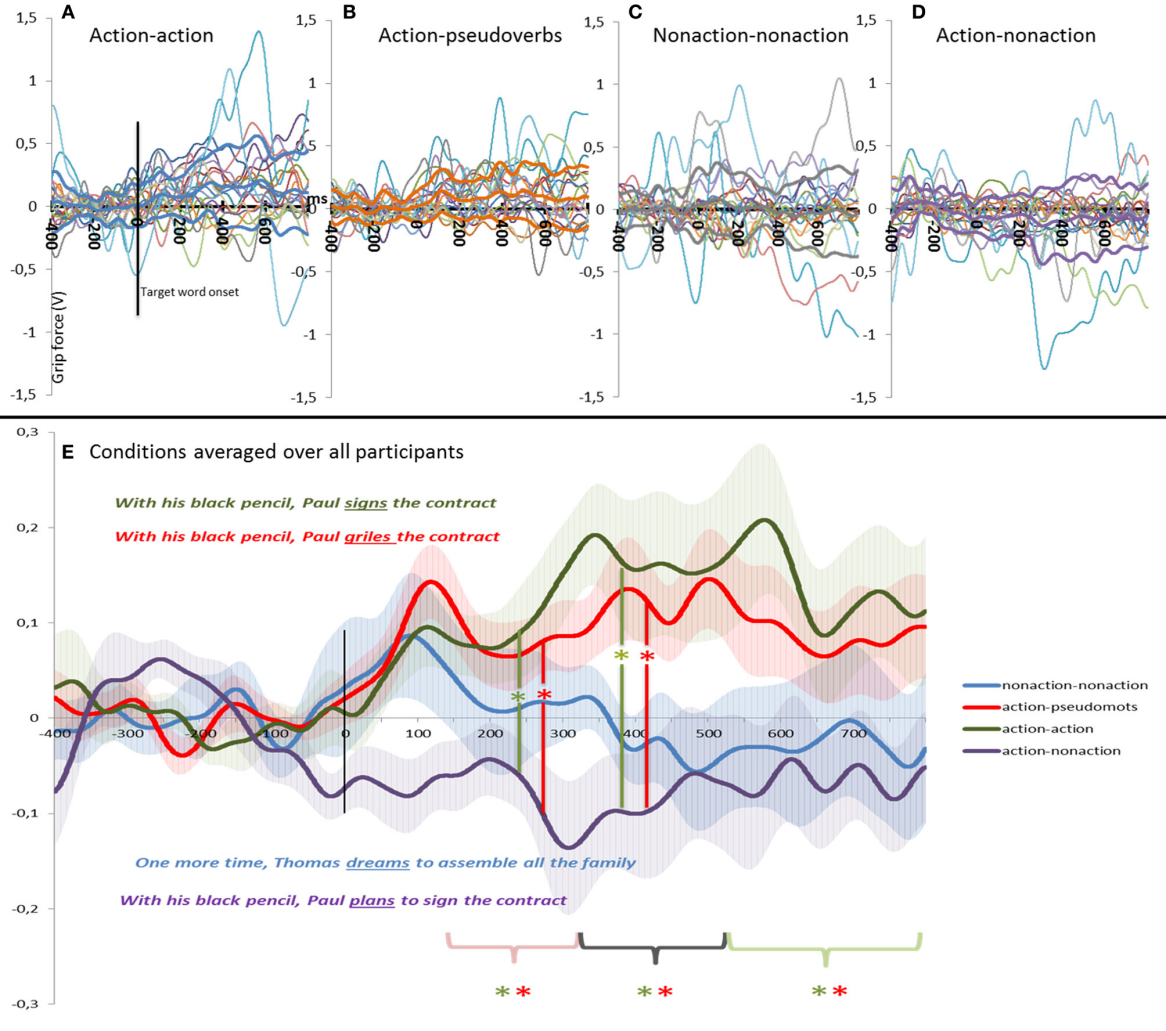
**Modulation of the grip-force amplitude as a function of time after target onset in Experiment 2 (Pseudo-verbs)**. **(A–D)** Displays individual data for the four conditions (the bold lines represent the means and standard deviations) and **(E)** compares data of the four conditions averaged over all participants. In **(E)** we also show the standard error of the mean (s.e.m.) around the mean value across the subjects (shaded regions). For the action-action condition and the action-pseudo-verb condition, a significant increase in the grip force was early observed, and maintained until the last interval. This enhanced grip-force is significantly different from action-non-action condition in the two first intervals. The color of the asterisk refers to the color of the condition that is compared.

Figure 3 plots the variations in grip-force amplitude as a function of time after target word onset for the four experimental conditions (action-action condition, action-pseudo-verb condition, action-non-action condition, and non-action-non-action condition). The top panel displays individual data for the four conditions and the bottom panel compares data of the four conditions averaged over all participants. As is obvious from the figure, for the action-action condition and the action-pseudo-verb condition, a steady increase in the grip force [the compression force component of the load cell (Fz)] was early observed, and maintained until the last interval. By contrast, the action-non-action condition appeared to cause a drop in the grip-force. Finally, non-action-non-action condition remained nearly constant at baseline.

For the Action-Action condition, the test against the baseline revealed a significant increase in the grip-force in the three time windows [*p* = 0.01, *p* = 0.02, and *p* = 0.04 for 120–320, 320–520, 520–800 ms respectively]. For the Action-Pseudo-verb condition, the test against the baseline also revealed a significant increase in the grip-force in the three time windows [*p* = 0.01, *p* = 0.006, and *p* = 0.01, respectively]. No significant effects against baseline were observed for the non-action verbs in the action context or for the non-action-non-action condition. The ANOVA was significant in all time windows [*F*_(3, 54)_ = 4.558, *p* = 0.0064, *F*_(3, 54)_ = 5.2004, *p* = 0.0032, and *F*_(3, 54)_ = 3.251, *p* = 0.0287, for the first, second and third window, respectively]. Results of the *post-hoc* tests (Bonferroni) are plotted in Table 4.

**Table 4:** **Results of the *post-hoc* tests (Bonferroni) for the different contrasts**.

	**Act.— Action**	**Act.— Pseudoword**	**Non act.— Non action**
**TIME WINDOW 120–320 ms**			
Act.—Non action	*p* = 0.010	*p* = 0.019	*p* = 0.167
Act.—Action		n.s	n.s
Act.—Pseudoword			n.s
**TIME WINDOW 320–520 ms**			
Act.—Non action	*p* = 0.006	*p* = 0.029	n.s
Act.—Action		n.s	*p* = 0.135
Act.—Pseudoword			n.s
**TIME WINDOW 520–800 ms**			
Act.—Non action	*p* = 0.061	*p* = 0.123	n.s
Act.—Action		n.s	n.s
Act.—Pseudoword			n.s

The comparison of the three critical conditions (Action-Non-action vs. Action-Action and Action-Pseudo-verbs) revealed significant effects in the first two time windows. First time window: Action-Non-action condition (*M* = −0.1 V, *SD* = 0.19) differed significantly from the Action-Action (*M* = 0.099 V, *SD* = 0.15) [*p* = 0.01] as well as from the Action-Pseudo-verbs conditions (*M* = 0.08 V, *SD* = 0.13) [*p* = 0.019]. Second time window: Action-Non-action condition (*M* = −0.1 V, *SD* = 0.3) vs. Action-Action condition (*M* = 0.16 V, *SD* = 0.28) [*p* = 0.006] and vs. Action-Pseudo-verb condition (*M* = 0.12 V, *SD* = 0.16) [*p* = 0.029]. In the third time window the same tendency was also evident but the differences with the Action-Non-action condition did not reached significance: Action-Non-action condition (*M* = −0.11 V, *SD* = 0.3) vs. Action-Action condition (*M* = 0.16 V, *SD* = 0.34) [*p* = 0.061] and vs. Action-Pseudo-verb condition (*M* = 0.13 V, *SD* = 0.23) [*p* = 0.123]. By contrast, the comparison with the Non-action-Non action condition did not survive the Bonferroni correction for multiple comparison (all *p*'s > 0.05).

Table 5 summarizes the effect sizes (Cohen d) of the different comparisons. In all time windows large effect sizes were found for the difference between the Action-Action vs. Action Non-action conditions as well as between the Action-Pseudoword vs. Action Non-action conditions. In the second and third time windows medium to large effect sizes were also found between the Action-Action vs. Non-action Non-action conditions and between the Action-Pseudoword vs. Non-action Non-action conditions.

**Table 5:** **Cohen's d for the differences between the various conditions in the three time windows**.

	**Act.— Action**	**Act.— Pseudoword**	**Non act.— Non action**
**TIME WINDOW 120–320 ms**			
Act.—Non action	1.16	1.14	0.67
Act.—Action		0.09	0.33
Act.—Pseudoword			0.28
**TIME WINDOW 320–520 ms**			
Act.—Non action	1.02	1.05	0.39
Act.—Action		0.19	0.79
Act.—Pseudoword			0.81
**TIME WINDOW 520–800 ms**			
Act.—Non action	0.84	0.90	0.27
Act.—Action		0.10	0.84
Act.—Pseudoword			0.61

## Discussion

Our experiments were designed to explore the impact of local linguistic context on word-induced neural activation of motor structures. There are two main results of this study. First, compatible with previous findings (Taylor and Zwaan, 2008; Zwaan et al., 2010) our work shows that linguistic focus as defined by Taylor and Zwaan (2008) modulates language-induced motor activity. The presence of an action word in an utterance is not in itself sufficient to trigger a related motor activation (see also Raposo et al., 2009; Aravena et al., 2012; Schuil et al., 2013). Second, our data further shows that the linguistic surrounding and the knowledge of situation it sets up can be sufficient to activate the motor properties of a contextually expected action verb. The actual presence of a known action word is not necessary for the activation of motor structures (for similar results in pragmatic context, see Van Ackeren et al., 2012). Importantly, however, the very same context can nonetheless fail to trigger relevant motor activation if the tested lexical item is a familiar word that has no associated motor features. Hence, contextual expectations set up by a given utterance are not in themselves sufficient to supersede a lexical meaning that does not involve a motor content. On the basis of this evidence, we argue that language-induced motor activation is neither driven by purely context-free lexical meaning access nor the result of a fully post-lexical higher order operation. Rather, the activation of motor structure results from the dynamic interactions of available lexical and contextual information that take part in the online construction of a complex mental model associated with the processing of a sentence meaning.

In Experiment 1, we used the modal operator “vouloir” (to want) to manipulate the mode of access to a described action by shifting the linguistic focus toward the agent's attitude with respect to the action. “Modality” is a grammatical category that allows relativizing the validity of sentence meaning to a set of possible situations (Perkins and Fawcett, 1983). Agent-oriented modalities focus on the internal state of an agent with respect to the action expressed by a predicate (Bybee et al., 1994). Volition thus focalizes the sentence on the agent's attitude toward the action rather than on the action itself (Morante and Sporleder, 2012). Our results show that motor structures were only recruited when the action verb was the focus of the sentence meaning and not when the sentence meaning focused on the agent's attitude toward the action. These findings are consistent with the linguistic focus hypothesis proposed by Taylor and Zwaan (2008) (see also Zwaan et al., 2010; Gilead et al., 2013). However, our study goes beyond what these authors found. Recall that Taylor and Zwaan (2008) showed that language-induced motor activation could “spill-over” from the actual action word to the linguistically adjacent post-verbal adverb, provided that the adverb modified the action. Our study goes further than these results because we show that motor activation for the *action word itself* can be switched on and off as a function of the linguistic focus. Critically, our study also provides the timing of the contextually constrained word induced motor activation: linguistic focus modulates motor activity within a temporal window that has been associated with lexical semantic retrieval (i.e., 300–500 ms after word onset, see Friederici, 2002).

The results of our first experiment thus suggest that the processing of an action verb can rapidly activate motor features of a denoted action. However, these motor features are only recruited when the denoted action is *relevant* within the currently elaborated situation model. The sensitivity of language-induced motor activation to the relationship between context and lexical semantics suggests that motor structures could serve semantic specification.

The findings of Experiment 2 show that word induced motor activation involves an early evaluation of the context against which the relevance of the action features of the potential verbs are determined (for studies on the anticipatory referential interpretation see, e.g., Kako and Trueswell, 2000; Kamide et al., 2003; Chambers and Juan, 2008; Bicknell et al., 2010). Our sentences were designed so that a fronted adverbial phrase and the subject of the sentence set up a situation in which a hand action was anticipated (i.e., the action context). Following this sentential context the ensuing verb was either a verb denoting a hand action, a verb denoting non-action, or a pseudo-verb unknown to the subject. As expected, when the verb denoted a hand action, an increase of grip force was observed shortly after word onset. Critically, grip force also increased with a pseudo-verb unknown to the listener, but not when a known verb with no motor denotation was presented instead (e.g., “With his black pen, James **plans** to …”). These data clearly testify that the increase of grip force was not merely an effect of context. One plausible explanation for our finding is that when a sentence contains an unknown word, the process of meaning construction fills the semantic gap with the most adequate content within the given context (in our case an action performed with the hand) until more information is available. In other terms, the listener maintains the situation model elaborated from previous context and integrates the unknown word into this representation. In our experiment, the instrument described in the adverbial phrase as well as the human agent (i.e., “With his black pen, James… ”) anticipate hand-action relevant motor features. By integrating this information the listener models a situation that foresees a particular action as a plausible thematic relation. When the ensuing verb is unknown to the listener the elaborated situation model is maintained and motor structures are recruited. However, when the ensuing verb is a known word that does not refer to an action, the non-action verb updates the modeled situation and cancels action representation anticipated by the context. Thus, contextual parameters might be understood as part of a representational state that is constantly restructured and revised following incoming information (see also McRae et al., 2005; Bicknell et al., 2010; Matsuki et al., 2011).

The results of our second experiment thus suggest that the construction of a situation model allows making rapid inferences and predictions for the elaboration of linguistic meaning. The brain generates a continuous stream of multi-modal predictions and pattern completion based on previous experiences (see, for example, Barsalou, 2009). This drive to predict is a powerful engine for online language comprehension (Federmeier, 2007; Elman, 2009).

In conclusion, together with our previous findings (Aravena et al., 2012) the present results indicate that the recruitment of motor structures during the processing of an action word hinges on specific conditions: (i) the context must focus on a motor action and (ii) the tested word form must not be *incompatible* with a contextually anticipated action, i.e., it has to be either compatible or neutral as in the case of a pseudo-verb. Hence, the processing of an action word does not recruit motor structures constantly. The same action word form that provokes motor activity in one linguistic context will cease to do so in another one. Note further that in conditions in which word processing recruits motor structures, this language-induced motor activity is observed within the time frames in which lexical meaning are believed to be retrieved (Friederici, 2002; Swinney and Love, 2002).

Although an increasing number of recent studies has started to account for the context dependency of motor activity (e.g., Sato et al., 2008; Rueschemeyer et al., 2010; Mirabella et al., 2012; Papeo et al., 2012; Tomasino and Rumiati, 2013) the majority of research programs are still strongly rooted in a “dictionary-like” perspective of word meaning (see Elman, 2004, 2011; Evans, 2006; Evans and Green, 2006 for critical reviews). The novelty of our work resides in the explicit integration of a theoretical and experimental framework that could serve to link current models of sentence processing to neurobiological data on action-meaning representation. The here observed on/off switching of motor activity with a given lexical item could be interpreted as evidence against the assumption that motor activity is necessarily a relevant part of the action word meaning (see also Schuil et al., 2013). If motor semantic features were indeed accessed via a modular, exhaustive and context-independent process (c.f. Swinney and Love, 2002) motor structures should be recruited in a consistent and mandatory manner. This, however, is clearly not the case. Yet, “low level” lexical semantic process and “higher level” processes of meaning integration are not serial, discrete, and encapsulated operations (for other examples concerning semantics as well as syntax see Friston, 2003; Kamide et al., 2003; McRae et al., 2005; Chambers and Juan, 2008; Bicknell et al., 2010; Matsuki et al., 2011; Papeo et al., 2012). Context can anticipate motor semantic features of lexical items (Experiment 2) and can also switch them off when they are not relevant within the situation model (Experiment 1). Findings like these question the notion that motor semantic features are “fixed parts” of the action word meaning (Hoenig et al., 2008; Raposo et al., 2009; Egorova et al., 2013; Tomasino and Rumiati, 2013). Note that even when a verb such as “open” is processed in isolation, comprehenders are likely to represent meaning by reference to some *frequently* encountered situation, e.g., opening a door or a bottle (see the situated concept representation proposed by Barsalou, 2003).

The question about the functional or epiphenomenal nature of motor structures in action-language processing might therefore not be put in terms of its participation to lexical semantics processing or to the construction of situation models. Rather, to determine the role of motor structures in language processes it is necessary to take into account the fact that language comprehension involves several sources of information that are elaborated in parallel and continuously adjusted to make sense of an utterance as it is perceived (Allwood, 2003; Cuyckens et al., 2003; Elman, 2011). Classical accounts of language-induced motor activity that sees language-induced sensorimotor activity either as epiphenomenon (Mahon and Caramazza, 2008; Hickok, 2009) or as integral part of word meaning (Glenberg, 1997; Barsalou, 1999; Pulvermuller, 1999) are both problematic in that they assume a model that endorses a fixed, dictionary-like set of lexical representations. The here-demonstrated rapidity, flexibility, and context dependency of language-induced motor activity to one and the same word are not compatible with such view. Rather, following Evans and Green (2006) and Elman (2011), we believe that words are “operators” that alter mental states (i.e., situation models) in context-dependent and *lawful* ways. If the timing under which an effect occurs is indicative of its source (lexical meaning or post-lexical) the early language-driven motor effects that we observed in our experiments allow suggesting that motor activity takes part in the action word meaning construction in conditions in which the action is in the linguistic focus.

In short, motor knowledge is part of the *meaning potential* of action words. It participates in the construction of meaning when a currently modeled situation focuses the action and might serve *meaning-specification*. It also allows prediction and pattern completion, which are important processes for fluent and efficient online language comprehension.

### Conflict of interest statement

The authors declare that the research was conducted in the absence of any commercial or financial relationships that could be construed as a potential conflict of interest.
