# Supplementary material for: Action relevance in linguistic context drives word-induced motor activity
Source: Front Hum Neurosci. 2014 Apr 1;8:163. doi: 10.3389/fnhum.2014.00163 (PMC3978346; doi:10.3389/fnhum.2014.00163)
Supplement: Supplementary file 1 [file Presentation1.PDF]

APPENDIX A : Sentences list Experiment 1.

Volition-in-focus Condition

1. Dans la menuiserie, Martin veut scier une planche de bois.
2. Dans le parc, Laurent veut jeter l'enveloppe par terre.
3. Dans la cuisine, Lucie veut râper des carottes pour la salade
4. Pour le piquenique, Timon veut saler les œufs durs
5. Dans la laverie, Celia veut tordre le linge pour l'égoutter
6. Dans la cour, Alice veut pincer la main de sa poupée
7. A la cantine, Elsa veut racler l'intérieur de la casserole.
8. Devant l'église, Lilian veut serrer la main du futur mari.
9. Dans la salle de prof, olivier veut signer la feuille d'évaluation
10. Dans l'atelier d'art, amandine veut vernir le coffre
11. Pour le petit déjeuner, Yvonne veut agiter la bouteille du lait
12. Dans sa chambre, Cannelle veut épiler ses bras
13. Au stade, Marion veut prendre son javelot gris
14. Devant son miroir, Prune brosse ses cheveux ondulés
15. A la plage, Cédric veut enfouir ses lunettes dans son sac
16. A la ferme, Robert ne fauche pas le blé de son champ
17. A la réunion, Delphine veut frapper sur la table avant de parler
18. Sur un banc, Hector veut gratter le dos de son chien
19. Dans la prison, Yannick veut griffer la main du gardian
20. Au cirque, Philippe veut jongler avec de massues
21. Sur le trottoir, Charles mendie avec son chapeau
22. Sur la carte, Eloïse veut montrer son pays d'origine
23. En coulisse, Sylvie veut peigner l'actrice principale
24. Dans la batucada, Nicolas veut secouer les maracas
25. Dans le pré, Greg veut arroser les tulipes
26. Dans son manoir, Harry veut balayer le plancher
27. Dans la salle de sport, Fiona veut soulever des haltères
28. Dans sa villa, Lionel veut astiquer la rampe d'escalier
29. À la crèche, Louise veut colorier la tête de son bonhomme
30. Devant la boite de nuit, Manon veut déchirer sa carte d'identité
31. Sur sa toile, Julien veut dessiner les nuages blancs
32. Devant son ordinateur, Richard veut Pianoter sur le clavier
33. Dans son bain, Léo veut savonner ses pieds
34. Sur son fauteuil, Claudia veut tricoter des chaussettes
35. Dans les magazines, Luc veut découper des images de maison

Action-in-focus condition

1. Dans le sentier, Jean scie un tronc d'arbre.
2. Dans la salle de classe, Bastien jette le papier dans la poubelle.
3. Pour le diner, Berta râpe du fromage dans ses pâtes.
4. Pour le barbecue, Abdala sale la viande.
5. A la piscine, Adela tord la serviette qui est tombé dans l'eau
6. A la fin du dîner, Abby racle le fond de son assiette.
7. Dans le magasin, Camille serre le nœud de ses chaussures.
8. Au bureau, Carlo signe le contrat.
9. Dans le magasin d'antiquités, Danielle vernit la table.
10. Dans la rue, David agite la main pour saluer.
11. A l'institut de beauté, Elena épile les jambes de sa cliente.
12. Au concert, Elias prend le microphone
13. Dans la salle de bain, Fabian brosse ses dents
14. Dans cette caverne, Fanny enfouit les objets précieux
15. Dans le jardin, Gaël fauche les mauvaises herbes.
16. A l'entrée de la maison, Gabrielle frappe la porte.
17. Dans l'atelier, Irène gratte la peinture qui a débordé.
18. Avec un costume de chat, Ian griffe le sol.
19. Dans les fêtes d'anniversaire, James jongle avec les oranges.
20. Dans le métro, Joseph mendie un morceau de pain
21. Par la fenêtre, Jacqueline montre le chemin.
22. Le matin, Mathilde peigne ses longs cheveux.
23. Dans le bar, Anne secoue la bouteille de jus.
24. Le soir, Vicente arrose les plantes.
25. En fin de journée, Karine balaye le trottoir.
26. A l'intérieur de l'avion, Laure soulève son bagage.
27. Dans la cuisine, Madeleine astique le dos de la casserole
28. Dans la maison de sa grand-mère, Stéphane colorie les dessins
29. A la poste, Maël déchire l'enveloppe de la lettre reçue.
30. A la campagne, Rémi dessine le contour des montagnes.
31. Dans les embouteillages, Patrick pianote sur le volant.
32. Dans la douche, Pauline savonne les cheveux de son enfant
33. Cet hiver, Sabine tricote une écharpe.
34. A l'école, Salvador découpe des personnages en papier.
35. Dans sa chambre, Mathilde peigne sa poupée.

Nouns condition

1. Dans la montagne, Léonard voit l'aigle qui plane.
2. Dans le bois, Arthur contemple le hêtre qui date de 1780.
3. Ce soir, Allan attend son avion pour aller en Écosse

4. Sur la rive, Frank choisit un canoë pour se promener.
5. Aujourd'hui, Aurélie découvre la grotte où est le trésor
6. Dans le ciel, Willy regarde une étoile filante très lumineuse.
7. Au zoo, Brigitte admire la toison fauve du tigre
8. De sa fenêtre, Chloé apprécie le mûrier en face de la cabane.
9. A l'aquarium, Damien observe le requin blanc
10. A la fin de la promenade, Daniel aperçoit le canyon du regard
11. A l'unanimité, Raphaël ouvre l'écluse au bateau.
12. Sur la colline, Aurore cherche le moulin le plus grand.
13. Par téléphone, Emma réserve la chambre d'hôtel
14. Chez le notaire, Erick estime le terrain à sa valeur actuelle
15. Dans le centre commercial, Léa inspecte la vitrine avant d'entrer
16. Dans la forêt, Emile explore le sentier embroussaillé
17. Dans le désert, Abdallah vénère son chameau.
18. Au printemps, Edmonde aime le bosquet en fleurs de son jardin
19. Dans le parc d'attraction, Thierry visite la caverne du dragon
20. Pendant la descente, Eléonore pense à la falaise derrière elle.
21. En Patagonie, Françoise étudie le fameux iceberg géant.
22. Dans son lit, Véronique rêve d'une licorne qui joue sur la pelouse
23. A la ferme, Victoria prend soin du pommier de sa grand-mère.
24. Dans ses rêves, Virginia imagine une prairie paisible.
25. Deux ans plus tard, Paul se rappelle de la tempête qui a frappé le sud.
26. Au fond du jardin, Yves a une oseraie très étendue
27. Au magasin, Sylvain achète un grillage pour son pré.
28. Quand il fait froid, Baptiste se souvient de la banquise de l'antarctique.
29. De la réserve, Antonin surveille la barrière de l'entrée.
30. Dans la maison, Nathan regarde la moquette du séjour.
31. Dans son appartement, Ophélia partage la penderie avec sa colocataire.
32. Dans sa maison de vacances, Oscar a besoin d'une rambarde pour les escaliers.
33. Dans son quartier, Raoul maudit le monument de la place.
34. Avant de mourir, Ryan lègue le cerisier à sa fille.
35. Finalement, Tara obtient le chevalet le plus haut.

English approximate translation

Volition-in-focus Condition

1. In the joinery, Martin wants to saw a wooden plank.
2. In the park, Laurent wants to throw the envelop on the ground.
3. In the kitchen, Lucie wants to grate carrots for the salad.
4. For the picnic, Timon wants to salt the hard-boiled eggs.
5. In the launderette, Celia wants to wring the cloth out.

6. In the yard, Alice wants to pinch her doll's hand.
7. In the canteen, Elsa wants to scrape the inside of the saucepan.
8. In front of the church, Lilian wants to shake the future husbands' hand.
9. In the teachers' staffroom, Olivier wants to sign the evaluation sheet.
10. In the art studio, Amandine wants to varnish the chest.
11. For breakfast, Yvonne wants to shake the bottle of milk.
12. In her bedroom, Cannelle wants to wax her arms.
13. At the stadium, Marion wants to take her grey javelin.
14. In front of her mirror, Prune wants to brush her wavy hair.
15. At the beach, Cédric wants to bury his glasses in his bag.
16. At the farm, Robert wants to mow the wheat of his field.
17. At the meeting, Delphine wants to hit the table before she speaks.
18. On a bench, Hector wants to scratch his dogs' back.
19. In the prison, Yannick wants to scratch the warder's hand.
20. At the circus, Philippe wants to juggle clubs.
21. On the sidewalk, Charles begs for money with his hat.
22. On the map, Eloïse wants to show her home country.
23. Behind the scenes, Sylvie wants to comb the leading actress.
24. During the batucada, Nicolas wants to shake the maracas.
25. In the meadow, Greg wants to water the tulips.
26. In his manor, Harry wants to sweep the floor.
27. At the gym, Fiona wants to lift the dumbbells.
28. In his villa, Lionel wants to polish the banister.
29. At the nursery, Louise wants to color the head of the man she drew
30. In front of the night club, Manon wants to tear her ID up.
31. On his canvas, Julien wants to draw white clouds.
32. In front of his computer, Richard wants to tap away on the keyboard.
33. In his bathtub, Léo wants to soap his feet.
34. In her armchair, Claudia wants to knit socks.
35. In magazines, Luc wants to cut house images out.

Action-in-focus condition

1. On the path, Jean saws a tree trunk.
2. In the classroom, Bastien throws the paper in the dustbin.
3. For dinner, Berta grapes cheese in the pasta.
4. For the barbecue, Abdala salts the meat.
5. At the swimming pool, Adela wrings the towel that had fallen in the water.
6. At the end of dinner, Abby scrapes the bottom of her plate.
7. In the shop, Camille tightens her shoe laces.
8. At work, Carlo signs the contract.
9. In the antiques shop, Danielle varnishes the table.

10. In the street, David waves the hand to say hello.
11. At the beauty institute, Elena waxes her customer's legs.
12. At the concert, Elias takes the microphone.
13. In the bathroom, Fabian brushes his teeth.
14. In this cave, Fanny buries precious objects.
15. In the garden, Gaël mows the weed.
16. At the house entrance, Gabrielle knocks on the door.
17. In the workshop, Irène scrapes the peint that was spilt.
18. With a cat costume, Ian scratches the floor.
19. In birthday parties, James juggles oranges.
20. In the subway, Joseph begs for a piece of bread.
21. Through the window, Jacqueline shows the path.
22. In the morning, Mathilde combs her long hair.
23. In the bar, Anne shakes the bottle of juice.
24. In the evening, Vincente waters the plants.
25. In the late afternoon, Karine sweeps the sidewalk.
26. Inside the plane, Laure lifts her luggage.
27. In the kitchen, Madeleine polishes the back of the saucepan.
28. In his grand-mother's house, Stéphane colors the drawings.
29. At the post office, Maël tears the envelop of the received letter up.
30. In the countryside, Rémi draws the outline of the mountains.
31. In the traffic, Patrick drums his fingers on the wheel.
32. In the shower, Pauline soaps her child's hair.
33. This winter, Sabine knits a scarf.
34. At school, Salvador cuts paper men up.
35. In her bedroom, Mathilde combs her doll's hair.

Nouns condition

1. In the mountain sky, Léonard sees the eagle gliding.
2. In the woods, Arthur contemplates the beech dating from 1780.
3. Tonight, Allan awaits his plane to go to Scotland.
4. On the riverbank, Frank chooses a canoe for the day.
5. In the sky, Willylooks at a bright shooting star.
6. Today, Aurélie discovers the cave where the treasure is hidden.
7. At the zoo, Brigitte admires the fleece of the fawn lion.
8. From her window, Chloé appreciates the mulberry tree facing the cabin.
9. At the aquarium, Damien observes the white shark.
10. At the end of the walk, Daniel sees the canyon.
11. Unanimously, Raphaël opens the lock for the boat.
12. On the hill, Aurore looks for the biggest mill.
13. On the phone, Emma books the hotel room.

14. At the solicitor's office, Erick estimates the value of the site.
15. In the shopping center, Léa inspects the shop window before walking in.
16. In the forest, Emile explores the bushy path.
17. In the desert, Abdallah venerates his camel.
18. In spring, Edmonde likes her garden's fower grove.
19. In the theme park, Thierry visits the dragon cave.
20. During the descent, Eléonore thinks about the cliff behind her.
21. In Patagonia, Françoise studies the famous giant iceberg.
22. In her bed, Véronique dreams about a unicorn playing in the grass.
23. At the farm, Victoria takes care of her grand-mother's apple tree.
24. In her dreams, Virginie imagines a peaceful meadow.
25. Two years later, Paul remembers the storm that hit the south.
26. In the back of the garden, Yves owns a vast rose garden.
27. In the shop, Sylvain buys a fence for his meadow.
28. When it is cold, Baptiste remembers the Antarctic ice field.
29. From the storeroom, Antonin watches the entrance gate.
30. In the house, Nathan looks at the living room fitted carpet.
31. In her apartment, Ophélia shares the wardrobe with her flatmate.
32. In his holiday house, Oscar needs a bannister for the stairs.
33. In the neighborhood, Raoul curses the historic monument.
34. Before he dies, Ryan bequeathes the cherry tree to his daughter.
35. Finally, Tara obtains the tallest easel.

APPENDIX B: Sentence list Experiment 2.

Action context – Action verb condition (A)

1. Avec ses beaux outils, Jean scie de fines planches de bois.
2. En un mouvement rapide de la main, William jette le papier à la poubelle.
3. Sur son clavier, Anne tape une lettre de motivation.
4. Avec un balai, Chloé bat le tapis persan.
5. De ses deux mains, Marc tord la serviette qui est tombée à l'eau.
6. Avec ses deux doigts, Alex pince le bras de sa camarade de classe.
7. A l'aide d'une cuillère, Claire racle le fond de la casserole.
8. Grâce à une clé anglaise, Anna serre un boulon sur son vélo.
9. Avec son stylo noir, Paul signe le contrat de renouvellement.
10. Avec son pinceau brosse, Thomas vernit le meuble ancien.
11. De ses deux bras, Diane agite le drapeau pour appeler à l'aide.
12. Avec une petite pince, Emma s'épile les jambes pour l'été.
13. Avec des gants de caoutchouc, Pierre prend le mollusque gluant.
14. Avec son arc, Lucas tire sur la cible.
15. A grands coups de pelle, Laure enfouit son trésor au fond du jardin.

16. Munie de sa serpette, Elise fauche les mauvaises herbes avec son père.
17. A l'aide de son marteau, Louis frappe sur le clou à plusieurs reprises.
18. Avec l'éponge, Alain gratte l'assiette sale jusqu'à ce qu'elle brille.
19. A l'aide d'une carafe, Jeanne verse de l'eau dans les verres.
20. D'une seule main, Irène jongle avec quatre balles.
21. Avec sa brosse rose, Lyse peigne les cheveux de sa Barbie avec soin.
22. Avec un shaker, Julie secoue les ingrédients pour préparer un cocktail.
23. A grands coups de balai-brosse, Bruno balaye le plancher de son manoir.
24. Grâce à un cric, Maud soulève la voiture pour changer le pneu crevé.
25. Avec un vieux chiffon, Marie astique le coffre de sa grand-mère.
26. Avec ses beaux feutres, Yann colorie les animaux de la ferme.
27. D'un coup de coupe-papier, Henri déchire l'enveloppe de la lettre tant attendue.
28. A l'aide de ses crayons de couleurs, Brice dessine un volcan en éruption.
29. De ses dix doigts, Nina pianote sur la table au rythme de sa chanson préférée.
30. Avec un gant de toilette, Steve savonne son enfant avant de le mettre au lit.
31. Equipée de ses longues aiguilles, Maxime tricote une écharpe rouge.
32. A l'aide de ciseaux, Sonia découpe des personnages en papier.
33. Avec son stylo à plume, Rose écrit une belle lettre à son amoureux.
34. Du bout du doigt, Max appuie sur le bouton rouge.
35. A l'aide de la bonne clé, Jacques ouvre le placard.
36. Avec un rouleau à pâtisserie, Jade aplatit la pâte à tarte.
37. A l'aide de grands couverts, Arthur remue la salade verte.

Action context – Non action verb condition (B)

1. A l'aide d'une scie électrique, Alain répugne à scier un tronc d'arbre.
2. En un geste rapide, Lucas feint de jeter la feuille à la poubelle.
3. Avec sa raquette de tennis, Maud s'applique à taper dans la balle.
4. Avec un batteur électrique, Emma rechigne à battre le beurre en crème.
5. Avec ses doigts, Marie peine à tordre une petite tige de fer.
6. Avec une pince, Anne se lasse de pincer les fils électriques.
7. Avec une fourchette, Bruno aspire à racler le fond de la casserole.
8. A l'aide d'une tenaille, Julie choisit de serrer le boulon qui bouge un peu.
9. Un crayon à la main, Rose se résout à signer le contrat sans le lire.
10. Par petites touches de pinceau, Elise s'ingénie à vernir ses ongles en bleu turquoise.
11. Dans la bouteille, Chloé pense agiter la vinaigrette avant de la verser sur sa salade.
12. A l'aide d'une crème dépilatoire, Louis consent à s'épiler le dos.
13. A travers ses moufles, Jean tâche de prendre de la neige pour en faire une boule.
14. Avec son revolver, Thomas projette de tirer sur des bandits en fuite.
15. A l'aide d'une pioche, Sonia hésite à enfouir son butin en plein jour.
16. A l'aide d'une faux, Henri rage de faucher les blés à l'ancienne.
17. D'un coup de poing, Steve essaye de frapper son adversaire en plein visage.

18. Avec ses ongles, Diane se résigne à gratter le fond de son assiette.
19. A l'aide de l'arrosoir, Max prévoit de verser de l'eau sur les plantes.
20. Avec huit balles de cirque, Maxime envisage de jongler une heure sans s'arrêter.
21. A l'aide d'un démêlant, Anna souhaite peigner ses cheveux crépus.
22. A l'aide de couverts en bois, Lyse se tâte à secouer la salade.
23. A l'aide d'un balai bleu, Nina décide de balayer la terrasse.
24. D'un seul bras, Brice aime soulever la grosse valise de sa femme.
25. Avec une brosse spéciale, Arthur ambitionne d'astiquer le parquet de son salon.
26. A l'aide de ses crayons de couleur, Yann rêve de colorier les dessins de son cahier.
27. D'un geste brusque de la main, Alex tente de déchirer son vieux jean.
28. Avec ses beaux feutres, Laure prône de dessiner ce qu'elle voit par la fenêtre.
29. Sur un clavecin noir, William compte pianoter une ancienne ritournelle.
30. Avec du gel douche, Claire se propose de savonner les pieds de ses enfants.
31. Dans son cours de tricot, Irène songe à tricoter des chaussettes.
32. Avec un couteau pointu, Jade désire découper son morceau de viande.
33. Avec un crayon à papier, Marc s'apprête à écrire des pense-bêtes sur des post-it.
34. Sur le bouton vert, Paul prétend appuyer de toutes ses forces.
35. D'un tour de poignée, Jacques daigne ouvrir la porte du grenier.
36. Du bout du doigt, Jeanne croit aplatir l'ourlet de son pantalon.
37. Avec une grande cuillère, Pierre conçoit de remuer la pâte à gâteau.

Action context -Pseudo verbs condition (C)

1. A l'aide d'une tronçonneuse, Bruno plucotte les arbres marqués d'une croix rouge.
2. D'un seul bras, Rose enfoupe son adversaire à terre.
3. Avec son poing, Anne hésipère à la porte pour qu'on lui ouvre.
4. Avec un fouet, Jade pièpe les blancs d'œufs en neige.
5. A grands coups de maillet, Jeanne gâne le clou, qui devient inutilisable.
6. Avec une pincette, Alain tellule les feuilles de la partition.
7. A l'aide d'une spatule, Thomas tasempe la nourriture collée au fond du bol.
8. Avec un tournevis, Jacques dève les vis permettant de sa construction.
9. D'un tracé de plume, Henri prache une lettre écrite sur parchemin.
10. Avec un vieux chiffon, Diane sange le meuble ancien.
11. D'un mouvement énergique de la main, Alex ésore la bouteille de jus.
12. Grâce à son épilateur électrique, Pierre se trasanne les jambes rapidement.
13. A l'aide de baguettes chinoises, Irène cétroche un sushi au saumon.
14. Avec une corde, Jean capame de l'eau du puits.
15. Avec une truelle, Lucas gricotte ses bien les plus précieux.
16. A coups de faucille, Max fanse les mauvaises herbes du jardin.
17. D'un coup de batte de baseball, Elise saude la balle qui parcourt plus de cent mètres.
18. A l'aide d'un grattoir, Chloé lore l'encre de chine qui déborde de sa lettre.
19. Avec la théière, Steve quopoud le thé dans les tasses en porcelaine.

20. Avec des boules multicolores, Maud caffre pour le plaisir de ses petits cousins.
21. Avec ses doigts, Marie haloque rapidement ses cheveux avant de sortir.
22. De ses deux mains, Maxime chencre le pommier pour en faire tomber les fruits.
23. A petits coups de balayette, Brice joine la chambre d'amis.
24. A l'aide d'un levier, Anna toupe la trappe qui mène au sous-sol.
25. Avec une brosse spéciale, Lyse britte le meuble ancien.
26. Avec des pastels, Yann achande les personnages de l'histoire.
27. En quelques traits de fusain, Arthur jotige un portrait de sa sœur.
28. Avec la déchiqueteuse, Marc vucle les contrats fallacieux.
29. Sur son synthé neuf, Nina épague en attendant son professeur de piano.
30. A l'aide d'un savon parfumé, Laure tassine ses mains.
31. Avec la technique du crochet, Louis salatit des chaussons pour son filleul.
32. A l'aide d'un cutter, Sonia shème des patrons en carton.
33. Muni d'un stylo à encre, Julie firre des poèmes dans son calepin.
34. Avec son pouce, Emma parmit sur la fenêtre pour l'ouvrir.
35. A l'aide d'un scalpel, Claire grile l'abdomen de son patient.
36. Avec un presse-papier, Paul vraite les feuilles qu'il veut ajouter à son herbier.
37. Grâce à une cuillère en bois, William commore les oignons qui cuisent dans la poêle.

Non action context – Non action verb condition

1. Cet après-midi, Lucas décide de se promener dans la campagne.
2. Au mois d'août, Nina adore se baigner dans la mer.
3. Tous les six mois, Elise daigne appeler ses grands-parents.
4. Comme tous les matins, Irène s'apprête à se regarder dans le miroir.
5. A l'aéroport, Anne se propose d'accueillir les voyageurs.
6. Dans le parc, Marc projette de rêvasser tout l'après-midi.
7. A onze heures du matin, Sonia aime faire une pause café.
8. Pour une fois, Steve consent à laisser la parole aux autres.
9. Dans l'après-midi, Arthur envisage de s'assoupir sur sa chaise longue.
10. Pour Pâques, Emma espère recevoir beaucoup de chocolat.
11. En hiver, Thomas déteste avoir froid.
12. Une fois de plus, Alain se résout à écouter au lieu de parler.
13. Par principe, Jade répugne à céder aux caprices de son fils.
14. Par moments, Laure conçoit d'oublier le travail.
15. Pour le petit-déjeuner, Brice choisit de rester au lit.
16. L'année prochaine, Yann ambitionne de suivre une formation d'ingénieur.
17. Au marché, Bruno hésite à acheter des carottes.
18. Devant le gendarme, Maxime prétend qu'on lui a volé ses papiers.
19. Pour ses enfants, Rose aspire à être la meilleure mère possible.
20. Pour les vacances, William pense naviguer sur le Nil.
21. Cette fois-ci, Julie accepte de considérer des études en médecine.

22. Avec tristesse, Paul se résigne à rentrer chez lui bredouille.
23. A cause de ces rumeurs, Maud se tâte à commander des plats chinois.
24. Régulièrement, Claire rêve de faire le tour du monde.
25. Pour son mari, Diane souhaite organiser une soirée d’anniversaire.
26. Pour le championnat de saut en hauteur, Lyse tente de passer la barre des 2 mètres.
27. Au bout de vingt ans de carrière, Alex songe à changer de profession.
28. La semaine prochaine, Jeanne compte demander une augmentation.
29. En rentrant de l’école, Marie désire raconter sa journée.
30. Pour le bal de fin d’année, Anna s’imagine danser toute la nuit.
31. Ce soir, Jacques prévoit de surprendre sa femme avec des fleurs.
32. Depuis plus d’un an, Jean cherche à entrer dans cette entreprise.
33. Le dimanche matin, Louis préfère regarder la télévision.
34. Cet après-midi, Max essaye de plaire à ses beaux-parents.
35. En observant son cousin, Chloé croit savoir ce qui le tracasse.
36. Le week-end, Henri a besoin de s’évader de son quotidien.
37. La veille de l’interrogation, Pierre s’applique à réciter sa poésie.

English approximate translation

Action context – Action verb condition (A)

1. With his beautiful tools, Jean saws thin wooden planks.
2. In a rapid movement of the hand, William throws the paper in the dustbin.
3. On her keyboard, Anne types a letter of motivation.
4. With a broom, Chloé beats the Persian carpet.
5. With his two hands, Marc wrings the towel that fell in the water.
6. With his two fingers, Alex pinches his classmate’s arm.
7. With a spoon, Claire scrapes the bottom of the saucepan.
8. With a monkey wrench, Anna tightens the bolt on her bicycle.
9. With his black pen, Paul signs the renewal contract.
10. With his paintbrush, Thomas varnishes the ancient piece of furniture.
11. With her two arms, Diane waves the flag to call for help.
12. With small pliers, Emma waxes her legs for summer.
13. With rubber gloves, Pierre takes the sticky mollusc.
14. With his bow, Lucas shoots at the target.
15. With a big shovel, Laure buries her treasure in the back of her garden.
16. With her pruning knife, Elise mows the weed with her father.
17. With his hammer, Louis hits the nail repeatedly.
18. With the sponge, Alain scrapes the dirty plate until it is shiny.
19. With a jug, Jeanne pours water in the glasses.
20. Single-handedly, Irène juggles four balls.
21. With her pink brush, Lyse combs her Barbie’s hair with care.
22. With a cocktail shaker, Julie shakes the ingredients of a delicious cocktail.

23. With a long-handled scrubbing brush, Bruno sweeps the floor of his manor.
24. With a jack, Maud lifts the car to change a puncture.
25. With an old cloth, Marie polishes her grand-mother's chest.
26. With his beautiful felt-tip, Yann colors the farm animals.
27. With a paper-knife, Henri tears the envelop of the long awaited letter.
28. Thanks to his color pencils, Brice draws an erupting volcano.
29. With her ten fingers, Nina drums on the table following her favorite song's rhythm.
30. With a flannel, Steve soaps his child before putting him to bed.
31. With long needles, Maxime knits a red scarf.
32. With scissors, Sonia cuts paper en up.
33. With her fountain pen, Rose writes a beautiful letter to her lover.
34. With the tip of his finger, Max presses the red button.
35. With the right key, Jacques opens the cupboard.
36. With a rolling pin, Jade flattens the pastry.
37. With big flatware, Arthur shakes the green salad.

Action context – Non action verb condition (B)

1. With an electric saw, Alain is reluctant to saw the tree trunk.
2. In a rapid gesture, Lucas pretends to throw the sheet in the dustbin.
3. With her tennis racket, Maud applies to hit the ball.
4. With an electric whisk, Emma balks at beating the butter into cream.
5. With her fingers, Marie struggles to twist a small rod.
6. With pliers, Anne grows tired of pinching electric wires.
7. With a fork, Bruno aspires to scraping the bottom of the saucepan.
8. With a pair of pincers, Julie chooses to tighten the loose bolt.
9. A pen in the hand, Rose resolves to sign the contract without reading it.
10. With small paintbrush strokes, Elise strives to varnish her nails in blue.
11. In the bottle, Chloé thinks about shaking the vinegar sauce before pouring it on the salad.
12. With a hair-removing cream, Louis agrees to wax his back.
13. Through his mittens, Jean tries to take the snow to shape it into a ball.
14. With his revolver, Thomas plans to shoot on the running bandits.
15. With a pickaxe, Sonia hesitates to bury her loot in broad daylight.
16. With a scythe, Henri fumes at the idea of mowing the wheat in the traditional way.
17. With a punch, Steve tries to hit his opponent in the face.
18. With her nails, Diane resigns herself to scraping the bottom of her plate.
19. With a watering can, Max plans to pour water on the plants.
20. With eight circus balls, Maxime considers juggling one hour straight.
21. With to a hair-conditioner, Anna wishes to comb her fuzzy hair.
22. With to wooden flatware, Lyse hesitates to shake the salad.
23. With to a blue broom, Nina decides to sweep the terrace.
24. With one arm, Brice likes to lift his wife's big luggage.
25. With a special brush, Arthur has the ambition to polish the living room floor.
26. With his color pencils, Yann dreams of coloring the drawings in his notebook.

27. With a sudden gesture of the hand, Alex attempts to tear his old jeans.
28. With her beautiful felt-tips, Laure recommends to draw what she sees through the window.
29. On a black harpsichord, William intends to tinkle away an old tune.
30. With a shower gel, Claire proposes to soap her children's feet.
31. In a knitting class, Irene thinks about knitting socks.
32. With a sharp knife, Jade wants to cut her loaf of meat.
33. With a black pencil, Marc gets ready to write reminders on post-its.
34. On a green button, Paul pretends to press with all his strength.
35. With a turn of the handle, Jacques deigns to open the attic door.
36. With the tip of her finger, Jeanne believes she is flattening her trousers hem.
37. With a big spoon, Pierre designs to stir the pastry.

Action context -Pseudo verbs condition (C)

1. With a chain saw, Bruno plucottes the trees that are marked with a red cross.
2. With one arm, Rose enfoupes her opponent to the ground.
3. With her fist, Anne hesiperes on the door for someone to open it.
4. With a whisk, Jade piepes the eggs whites until stiff.
5. With heavy mallet blows, Jeanne ganes the nail, making it unusable.
6. With a pair of tweezers, Alain tellules the score pages.
7. With a spatula, Thomas tasempes the food stuck at the bottom of the bowl.
8. With a screwdriver, Jacques deves the screws allowing for the construction.
9. With a nib, Henri praches a letter on parchment.
10. With an old cloth, Diane sanges the old piece of furniture.
11. With a dynamic hand gesture, Alex esores the juice bottle.
12. With his electric epilator, Pierre trasames his legs quickly.
13. With chopsticks, Irène cetroches a salmon sushi.
14. With a rope, Jean capames water from the well.
15. With a trowel, Lucas gricottes his most precious goods.
16. With a sickle, Max fanses the garden weed grass.
17. With a baseball bat blow, Elise saudes the ball, which covers over a hundred meters.
18. With a scraper, Chloé lores the Indian ink overflowing her letter.
19. With the teapot, Steve quopouds the tea in porcelain teacups.
20. With multicolored balls, Maud caffres to amuse her little cousins.
21. With her fingers, Marie quickly haloques her hair before going out.
22. With his two hands, Maxime chencres the apple tree to make the fruits fall.
23. With small brush strokes, Brice joines the guest room.
24. With a lever, Anna toupes the trap door leading to the basement.
25. With a special brush, Lyse brittes the ancient piece of furniture.
26. With pastels, Yann achandes the great men of history.
27. With a few lines of charcoal, Arthur jotiges a portrait of his sister.
28. With the shredder, Marc vucles the fallacious contracts.
29. On her new synthesiser, Nina epagues while waiting for her piano teacher.
30. With a perfumed soap, Laure tassines her hands.

31. With the crochet technique, Louis salatits slippers for his godchild.
32. With a cutter, Sonia shemes sewing patterns in cardboard.
33. With an ink pen, Julie firres poems in her notebook.
34. With her thumb, Emma pirmits on the window to open it.
35. With a scalpel, Claire grittes the abdomen of her patient.
36. With a paperweight, Paul vraites the leaves he wants to add to herbarium.
37. With a wooden spoon, William commores the onions that are cooking in the pan.

Non action context – Non action verb condition

1. This afternoon, Lucas decides to take a walk in the country.
2. In August, Nina loves to bathe in the sea.
3. Every six months, Elise calls her grand-parents.
4. Every morning, Irene gets ready to look at herself in the mirror.
5. At the airport, Anne offers to welcome the travelers.
6. In the park, Marc plans to daydream all afternoon.
7. At eleven in the morning, Sonia likes to take a coffee break.
8. For once, Steve agrees to letting others speak.
9. In the afternoon, Arthur envisages to fall asleep in his deckchair.
10. For Easter, Emma hopes to receive a lot of chocolate.
11. In winter, Thomas hates to be cold.
12. One more time, Alain resolves to listen instead of speaking.
13. On principle, Jade is reluctant to give in to her son's whims.
14. From time to time, Laure plans to forget about her work.
15. For breakfast, Brice chooses to stay in bed.
16. Next year, Yann has the ambition to follow an engineering course.
17. At the market, Bruno hesitates to buy carrots.
18. In front of the policeman, Maxime pretends he was stolen his papers.
19. For her children, Rose aspires to be the best mother.
20. For the holidays, William thinks about sailing the Nile.
21. This time, Julie accepts to consider studies in medicine.
22. With sadness, Paul resigns himself to go home empty-handed.
23. Because of the rumors, Maud hesitates to order the Chinese dishes.
24. On a regular basis, Claire dreams of traveling around the world.
25. For her husband, Diane wishes to organize a birthday party.
26. For the high-jump championship, Lyse tries to jump the 2 meters bar.
27. After a carrier of twenty years, Alex thinks about starting a new profession.
28. Next week, Jeanne plans to ask for a raise.
29. Back from school, Marie wishes to tell about her day.
30. For the prom, Anna imagines herself dancing all night.
31. Tonight, Jacques plans to surprise his wife with flowers.
32. Since last year, Jean tries to enter this company.
33. Sunday morning, Louis prefers to watch television.
34. This afternoon, Max tries to please his parents-in-law.

35. While observing her cousin, Chloé thinks she knows what is bothering him.
36. On weekends, Henri needs to get away from his routine.
37. The day before the test, Pierre applies to recite his poem.

APPENDIX C: Parameters of lexical control.

VERBS	frequency ranges	Letters	Syllables	Bigrams	Trigrams
scier	2,39	5	1	2053,7	232,24
jeter	38,77	5	2	6096,66	563,97
râper	0,23	5	2	1759,06	99,85
Saler	0,39	5	2	6306,76	471,06
Tordre	2,9	6	1	5814,48	338,09
Pincer	2,35	6	2	3354,96	277,8
Racler	1,06	6	2	3989,2	227,73
Serrer	13,42	6	2	8611,9	1106,28
signer	9,23	6	2	3330,94	544,8
vernir	0,39	6	2	3561,04	660,46
agiter	6,68	6	3	4791,1	466,91
épiler	0,68	6	3	3463,74	210,76
prendre	256,16	7	1	5136,04	955,6
brosser	1,65	7	2	4158,96	599,1
enfouir	1,9	7	2	4528,46	371,87
faucher	2,06	7	2	3594,3	728,2
frapper	21,19	7	2	2929,02	354,41
gratter	4,94	7	2	4152,75	744,68
griffer	1,39	7	2	2372,61	141,21
jongler	0,94	7	2	6503,16	289,8
mendier	1,81	7	2	4827,74	908,19
montrer	66,61	7	2	10581,79	2856,44
peigner	0,81	7	2	3148,86	288,22
secouer	8	7	2	5271,19	540,37
arroser	2,55	7	3	2497,37	412,32
balayer	4,19	7	3	2455,48	246,47
soulever	11,45	8	2	9276,43	1187,34
astiquer	1,16	8	3	3880,11	594,07
colorier	0,32	8	3	5898,38	615,55
déchirer	5,16	8	3	3705,59	572,54
dessiner	9,74	8	3	16644,66	3172,44
pianoter	0,19	8	3	2788,21	149,2
savonner	0,77	8	3	3341,17	403,38
tricoter	1,77	8	3	2900,61	193,54
découper	3,81	8	3	3043,4	486,1
	13,9	6,8	2,3	4765	629

NOUNS	frequency ranges	Letters	Syllables	Bigrams	Trigrams
aigle	9	5	1	3627,42	194,03
hêtre	3,1	5	1	4917,89	1667,96
avion	34,71	5	2	3791,62	237,67
canoë	1,29	5	3	4856,14	159,17
grotte	12,35	6	1	4013,74	424
étoile	32,42	6	2	3838,43	227,65
toison	3,42	6	2	8015,57	1263,44
mûrier	0,35	6	2	2879,87	288,44
requin	1,29	6	2	3741,16	159,44
canyon	0,58	6	2	4775,36	98,74
écluse	1,9	6	2	1672,2	184,56
moulin	14,52	6	2	11156,36	676,74
chambre	231,23	7	1	3132,07	1005,93
terrain	61,87	7	2	4704,97	969,53
vitrine	11,42	7	2	4474,5	532,88
sentier	16,39	7	2	7737,99	1324,38
chameau	3,52	7	2	3897,52	1058,85
bosquet	1,77	7	2	2248,54	599,46
caverne	4,9	7	2	2999,25	412,82
falaise	9,74	7	2	4701,2	798,53
iceberg	0,77	7	2	1188,83	31,97
licorne	1,1	7	2	2571,27	397,42
pommier	5,35	7	2	7236,32	1767,02
prairie	9,29	7	2	6623,51	663,49
tempête	17,42	7	2	2971,79	562,34
oseraie	0,29	7	3	2658,04	311,02
grillage	5	8	2	1899,71	319,69
banquise	1	8	2	3695,2	282,94
barrière	12,48	8	2	4371,53	391,89
moquette	7,97	8	2	2650,77	339,62
penderie	1,39	8	2	4693,45	765,49
rambarde	1,32	8	2	1494,19	156,91
monument	8,61	8	3	6753,73	1246,29
cerisier	1,68	8	3	6076,53	479,05
chevalet	3,35	8	3	2509,06	544,19
	15,2	6,8	2,0	4245	587

FQ OCCU	F(1, 142)=.0006; p = .9798	0,90
SYLL	F(1, 142)=1.7373; p = .1897	0,09
BIGR	F(1, 142)=1.8422; p = .1769	0,39
TRIG	F(1, 142)=.5321; p = .4670	0,76

	NOUNS	VERBS
FRQ	13,92	15,22
LETT	6,80	6,80
SYLL	2,26	2,03
BIGR	4765	4245
TRIG	629	587

APPENDIX D: Stimuli Validation

Action and non-action words validation

Frequency and the degree of effector specificity of action and nonaction words were controlled.
The frequency of use of target words was evaluated with the Lexique 3 data base (New et al., 2001). All target words presented moderate levels of frequency.
As a measure of "degree of effector specificity of action sentences", 36 subjects were asked to evaluate, on a 1 (this is not a hand action) to 5 (this is a hand action) rating scale, if the action encoded by the sentence was a hand action. All hand actions expressed in the action-action sentences were highly prototypical of their effector (*M*= 4.9, *SD*= 0.05, *M*= 4.8, *SD*= 0.08, *M*= 4.8, *SD*= 0.12 for A, B, and C action context lists, respectively).
To validate that non-action verbs denoted no action performed with the hand or arm we have considered as non-action verbs only those with low degree of effector specificity (under 2) (*M*= 1.1, *SD*= 0.18).

Action context validation

The three lists of action contexts were validated regarding the cloze probability of the hand action verb applying a questionnaire to 36 undergraduate students.
To determine whether context was predictive of the verb, subjects were asked to evaluate how fitting the final verb of the sentence was to the previous context using a 5-point Likert scale. Zero scores indicated that verbs were extremely unpredictable by their contexts and a score of 5 indicated high predictability. To ensure that context was predictive of the verb, sentences with low verb predictability (under 4) were eliminated (*M*= 4.46, *SD*= 0.22).
Pseudo-verbs validation

Thirty-seven pseudo-verbs were created obeying French's phonotactic constraints using the « Lexique Toolbox » of the data base Lexique 3 (New et al., 2001).
They were validated by applying a questionnaire to 36 undergraduate students about the soundness of the verb as a French verb. Subjects were asked to judge yes or no the pseudo-verb sound as a French verb. Pseudo-verbs with a score under 85% were eliminated (*M*= 93.6, *SD*= 4.4).
